# Case Report: HLA-DRB1 04:01 found in a child with adenovirus type 2 -linked hepatitis

**DOI:** 10.3389/fimmu.2025.1544622

**Published:** 2025-02-26

**Authors:** Yoshiki Katsumi, Yui Nishimura, Sachiko Goto, Seiichiro Ozawa, Tomoko Nishiura, Akira Kotera, Yoshiyuki Kawahara, Shiori Higashikawa, Rina Iwasaki, Yutaka Toriiminami, Norio Asai, Naohisa Fujita

**Affiliations:** ^1^ Department of Pediatrics, Kyoto Saiseikai Hospital, Kyoto, Japan; ^2^ Otokuni Health Center, Kyoto, Japan; ^3^ Kyoto Prefectural Institute of Public Health and Environment, Kyoto, Japan

**Keywords:** hepatitis, child, HLA-DR, adenovirus, common cold

## Abstract

Since 2022, cases of hepatitis of unknown origin have been reported in children worldwide. Adeno-associated virus type 2 (AAV2) was identified as a cause, with most affected children having the HLA-DRB1 04:01 genotype. In this study, we hypothesized that HLA-DRB1 04:01 in the host may also be a potential predisposing factor of acute hepatitis caused by other viruses. We report a case that met the definition of severe hepatitis of unknown cause in a child; adenovirus type 2 (AV2) was detected in her specimens. The patient was a 1-year-old girl who visited a doctor because of fever occurring 1–2 days per week, respiratory symptoms, and diarrhea. One month later, the patient was referred to our hospital because of prolonged elevated liver enzyme concentrations. Two weeks after the initial visit, her aspartate aminotransferase (AST) and alanine aminotransferase (ALT) concentrations increased to 1558 and 1843 IU/L, respectively. The patient’s liver enzyme concentrations decreased markedly with only observation and intravenous hydration during hospitalization within a few days. Thereafter, hepatic enzymes were transiently elevated with each common cold, but all recovered spontaneously. The adenovirus (AV) antibody levels increased substantially 2 weeks after admission. The patient’s human leukocyte antigen (HLA) was determined to be of the DRB1 04:01 genotype. The presence of HLA-DRB1 04:01 is consistent with that reported in pediatric patients with AAV2 hepatitis in the United Kingdom, indicating that it may have been involved in the host immune response and acute hepatitis in this child. HLA-DRB1 04:01 may predispose children to acute hepatitis from various viruses, including AV2, AAV2, and possibly respiratory viruses, which requires clinical attention.

## 1 Introduction

Since the initial reports of children with acute hepatitis of unknown origin in Scotland (1, 2), similar cases have been identified globally. Although adenovirus type 41 (AV41) or adeno-associated virus type 2 (AAV2) was suspected to be the cause of hepatitis in Europe and the USA (3, 4), no evidence was found.

The human leukocyte antigen (HLA) type has been suggested to affect hepatitis susceptibility via immune-modifying effects in hosts, and HLA-DRB1 04:01 has been reported as a host factor responsible for autoimmune hepatitis (AIH) in adults (5). Here, we report a case of acute severe hepatitis in which adenovirus type 2 (AV2) was identified and HLA-DRB1 04:01 was detected in the host; this genotype was suspected to be the immunological background for factors contributing to the development of hepatitis following the coronavirus disease 2019 (COVID-19) pandemic.

A 1-year-old girl first visited a local doctor due to recurrent fever (1–2 days a week), coughing, runny nose, and diarrhea, which persisted for 2 weeks after starting nursery school. The doctor consulted us due to a prolonged elevation of liver enzymes for a month, with aspartate aminotransferase (AST) ranging from 86 to 101 IU/L and alanine aminotransferase (ALT) from 110 to 185 IU/L. Upon arriving at our hospital, the patient’s body temperature was recorded to be 37.1°C, with no jaundice or hepatosplenomegaly observed. The patient appeared healthy and had no family history of hepatitis.

## 2 Case description

The liver enzyme concentrations changed negligibly until two weeks after the first visit to our hospital, when the patient’s AST and ALT concentrations suddenly increased to 1558 IU/L and 1843 IU/L, respectively, and gamma-glutamyl transpeptidase concentration was 188 U/L (**Table 1**). She was admitted as a case of severe hepatitis of unknown cause, many of which had been reported at that time. The patient’s temperature remained in the range of 37 to 38°C, and there was no jaundice, somnolence, vomiting, or abdominal pain. Abdominal computed tomography indicated mild hepatomegaly. We administered intravenous hydration to the patient after examination. Four days after admission, the patient was discharged from the hospital after spontaneous recovery and reduction of enzyme concentrations to 124 IU/L (for AST), 484 IU/L (for ALT), and 100 U/L (for gamma-glutamyl transpeptidase).

**Table 1:** Biological parameters of the patient in admission.

Parameters	Results
White blood cell (10^9^/L)	9.73
Neutrocyte (10^9^/L)	5.74
Lymphocyte (10^9^/L)	2.63
Hemoglobin (g/L)	117
Hematocrit (%)	36.4
Platelet (10^9^/L)	431
Aspartate aminotransferase (U/L)	1558
Alanine aminotransferase (U/L)	1843
Lactate dehydrogenase (U/L)	860
Prothrombin time (second)	11.3
Prothrombin time-international normalized ratio	1.07
Activated partial thromboplastin time (second)	47.0
Fibrinogen (mg/dL)	385
D-dimer (ug/mL)	0.5
Total protein (g/L)	70
Albumin (g/L)	40
Total bilirubin (umol/L)	6.84
Direct bilirubin (umol/L)	3.42
Leucine aminopeptidase (U/L)	225
gamma-glutamyl transpeptidase (U/L)	188
Total bile acid (umol/L)	107.2
Cholinesterase (U/L)	295
Amylase (U/L)	49
Ammonia (umol/L)	31.71
C-reactive protein (mg/dL)	0.25
Hepatitis A virus antibody	<0.50
Hepatitis A virus IgM antibody	negative
Hepatitis B virus surface antigen (IU/mL)	0.007
Hepatitis B virus DNA level (Log Iuml)	not detected
Hepatitis B virus e antigen	negative
Hepatitis B virus e antibody	negative
Hepatitis B virus c IgM antibody	negative
Hepatitis B virus c IgG antibody	negative
Hepatitis B virus surface antibody (mIU/mL) *	620
Hepatitis C virus antibody	0.2
Hepatitis E virus IgA antibody	negative
Lactate (mmol/L) †	0.99
Pyruvic acid (mmol/L) †	0.06
Immunoglobulin G (mg/dL)	1184
Alpha-feto-protein (ng/mL)	3.6
Antinuclear antibody	negative
Herpes symplex virus IgM antibody	negative
Herpes symplex virus IgG antibody	negative
Cytomegalovirus IgM antibody	negative
Cytomegalovirus IgG antibody	negative
Epstein-Barr virus VCA IgM antibody	negative
Epstein-Barr virus VCA IgG antibody	negative
Epstein-Barr virus-determined nuclear antigen	negative
Adenovirus antibody	negative
Measles virus IgM antibody	negative
Measles virus IgG antibody	negative
Rubella virus IgM antibody	negative
Rubella virus IgG antibody	negative
Adenovirus antigen §	positive
Respiratory Syncytial virus antigen §	negative
COVID19 virus antigen §	negative

The patient was negative for adenovirus antibody (<four-fold) at the time of admission but became positive (64-fold) after 2 weeks. Hepatitis B infection was ruled out based on the results of tests assessing other antigens, antibodies, and hepatitis B virus DNA; however, the test for hepatitis B virus surface antigen was slightly positive. *Three times of inactivated Hepatitis B virus vaccine have been injected. †The samples were collected from vein. §The samples were collected by nasopharyngeal swab.

## 3 Diagnostic assessment

When the patient was admitted to our hospital, pharyngeal swabs and blood, stool, and urine specimens were collected to identify the causative pathogen. Specimens were used for viral isolation, polymerase chain reaction (PCR), neutralizing antibody testing, and metagenomic analysis. All specimens were identified as AV2 by sequencing. Other hepatitis-causing viruses were not detected in PCR of the blood, stool, and throat swab specimens (**Supplementary Table 1**). The AV antibody level was less than four-fold at the time of admission and increased 64-fold subsequently, confirming acute adenovirus infection at the time of hepatitis. In the metagenomic analyses, human mastadenovirus C was detected in all specimens, which was consistent with the results of sequencing. No other causative bacteria or viruses, including AAV2, were detected (**Supplementary Figure 1**). Based on these results, the patient was diagnosed with hepatitis and acute AV2 infection. There was no history of vaccination or illness with severe acute respiratory syndrome coronavirus 2 (SARS-CoV-2), and the result of the SARS-CoV-2 antibody test was negative.

**Figure 1:**
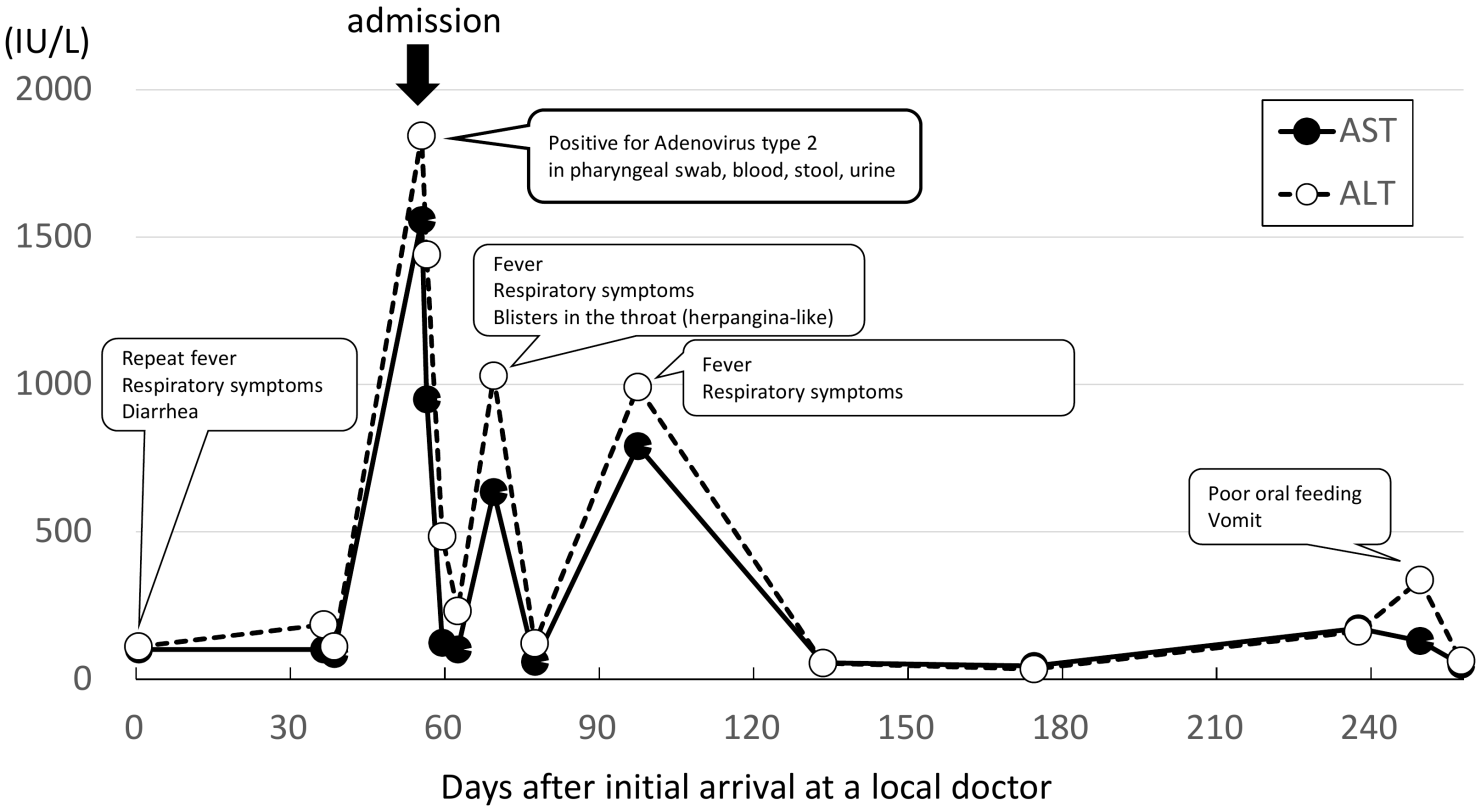
Symptoms and changes in liver enzyme concentrations. The patient went to a nursery school when she felt good and did not have a fever, suggesting that she might possibly have been infected with other pathogens after the adenovirus type 2 infection.

The patient’s HLA-DRB1 genotype was 04:01, which is consistent with the result of a previously-reported case of childhood hepatitis involving AAV2. Two weeks after discharge, the patient presented with symptoms of coughing, runny nose, fever, and blisters in the throat, similar to those seen in patients with herpangina but with no diarrhea. The patient presented elevated liver enzymes (highest AST 635 IU/L and highest ALT 1029 IU/L), and was again diagnosed as severe hepatitis of unknown cause, but the child recovered after a few days without hospitalization (**Figure 1**). At that time, only viral culture and PCR tests were performed for AV2 on blood samples. Cytopathic effects were observed in the third passage and AV2 was confirmed by sequencing. However, it was difficult to determine whether the result was truly AV2 infection or viral contamination. Another month later, fever and respiratory symptoms re-appeared, and elevated liver enzymes (highest AST 791 IU/L and highest ALT 991 IU/L) again met the definition of severe hepatitis of unknown cause. This child recovered without hospitalization. Only blood was used for viral culture and PCR testing, and nothing viral (including AV2) was detected. We did not perform a metagenomic analysis.

## 4 Discussion

From the present case, we inferred that the HLA-DRB1 04:01 genotype may be a factor that predisposes to acute hepatitis caused by AV2. Pediatric cases of acute hepatitis of unknown cause have been reported worldwide, including in Europe and the USA, starting with a report from Scotland in 2022 (1, 3, 6). AV41 and AAV2 are suspected to cause acute hepatitis (1, 3, 4). However, no definitive conclusions have been made regarding the causative viruses and underlying mechanism. A Japanese survey could not detect any trend that may suggest a causal relationship with AV41 or AAV2 (7). Fifty pediatric cases of hepatitis of unknown cause were analyzed from 2017–2023 in Japan (including the pre-COVID-19 pandemic period). The number of AAV2-positive cases was 12% (6 out of 49 cases) from PCR using blood samples. Co-infection with AV, human herpes virus 6, cytomegalovirus, or Epstein–Barr virus as helper viruses was also observed among AAV2-positive cases (half of the cases had co-infections). Hepatitis due to AAV2 appeared to have occurred sporadically before the COVID-19 pandemic (8).

Studies have shown that the whole blood of 90% children with acute hepatitis of unknown etiology was positive for adenovirus (3, 6). However, the number of AV-positive cases with acute hepatitis that harbored AV2 in this analysis was not clear. AV2 was the most frequent (26%) isolated AV in a Japanese survey in which all reported adenovirus infections were investigated from 2008–2017 (9), whereas AV2 was detected in very few cases of hepatitis after 2022 (10).

Does any host element increase the predisposition to hepatitis? Reports show that the HLA type is involved in the induction of hepatitis as a host factor in patients with AIH (5). AIH is a chronic form of hepatitis that is common in middle-aged women. The disease begins with fatigue, jaundice, and anorexia. Viral infections and some drugs are considered triggers of AIH; however, a genetic predisposition based on HLA is also thought to be involved. Reports from Japan, England, China, and Iraq suggest that HLA-DRB1 04:01 is associated with AIH type 1 in adults (5, 11–17). In Japan, 154 cases of AIH in adults aged 48–66 years were reported (201 cases in healthy subjects). The liver histopathology of adenovirus-associated hepatitis resembles that observed in AIH, suggesting the involvement of immune-mediated processes (18). Since various viral infections are proposed to trigger AIH (19), AV infection could also potentially trigger AIH. In the current case, a liver biopsy was not performed; it was therefore difficult to identify the cause of hepatitis as AV2 infection in the liver. Based on the acute course and the patient’s age, AIH was excluded from the diagnosis in this case.

HLA-DRB1 04:01 is found in less than 1% of the Japanese population. HLA-DRB1 04:01 has been reported in pediatric patients with AAV2-induced hepatitis in the United Kingdom (4). In this study, of the 27 cases of hepatitis with AAV2 infection in children, 25 (93%) had the HLA-DRB1 04:01 genotype. In contrast, 10 (15.6%) of the 64 controls (including 12 adenovirus-infected patients without elevated liver enzyme concentrations and 33 patients with elevated liver enzyme concentrations but without adenovirus infection) had the HLA-DRB1 04:01 genotype. According to the British Biobank, the frequency of HLA-DRB1 04:01 in the United Kingdom is 2942/29379 (10%).

Studies suggest that HLA class II genes, such as *HLA-DPB1* and *HLA-DRB1*, which are involved in presenting antigens to T cells, may influence the immune response to viruses and viral vaccines (20–23).

In the current patient’s last hepatitis episode, AV2 was not detected, but we could not rule out AV2 as the cause. The patient presented with upper respiratory tract inflammatory symptoms consistent with the common cold, suggesting a possible infection with a respiratory virus, such as rhinovirus. Human rhinovirus, which is the causative virus in 50% of common colds, infects vascular endothelium and initiates an antiviral and inflammatory innate response (24, 25). In Australia, the association between picornaviruses and respiratory syncytial virus and pediatric hepatitis was suspected before the COVID-19 pandemic, and the association between picornaviruses and pediatric hepatitis became stronger after the pandemic (26). Both human rhinovirus and enterovirus, which might have caused a common cold in the current case, are picornaviruses. This risk may be heightened when these viruses are present in larger quantities in the blood, due to reduced immunity against them caused by COVID-19 measures. It may not be necessary for the respiratory virus to infect hepatocytes; infection of endothelial cells within the liver can also cause liver damage. The liver damage observed in the current patient, associated with the upper respiratory tract infection, could be linked to the HLA-DRB1 04:01 genotype.

This was a single case report, and the possibility that the HLA-DRB1 04:01 genotype was incidentally detected in a host with hepatitis caused by AV2 cannot be ruled out. However, HLA-DRB1 04:01 is rare in Japan, found in less than 1% of the population. Based on this proposed mechanism, we assumed that host HLA-DRB1 04:01 was a possible predisposing factor for acute hepatitis caused by various viruses, including AV2, AAV2, and possibly respiratory viruses, although the causal relationship could not be confirmed.

In the future, it is expected that the immunological mechanism will be verified by basic research using HLA-DRB1 04:01 transgenic mice and viral infection of these mice; the causal relationship can then be elucidated using clinical multi-case studies of acute hepatitis in children analyzed for HLA and liver tissue histopathology.

## 5 Methods

To analyze the metagenome in pharyngeal swabs and blood, stool, and urine specimens, we extracted RNA using the QIAamp Viral RNA Mini Kit (QIAGEN, Redwood City, CA) and prepared the RNA library according to the protocol of the Zymo-Seq RiboFree Total RNA Library Kit (Zymo Research, Irvine, CA). Libraries were sequenced on the iSeq100 system or MiSeq system (Illumina, San Diego, CA) with 2 × 150 bp paired ends. The output FASTQ files were trimmed, and human-derived reads were removed using kneaddata v0.12.0 (https://github.com/biobakery/kneaddata). Using kraken2 v2.1.3 (27), the data were classified. The report was then visualized using krona v2.8.1 (28) to search for pathogens. HLA-DRB1 four-digit allele typing was performed using the Luminex 200 system (Luminex, Austin, TX) and the WAKFlow HLA Typing Kit (Wakunaga, Hiroshima, Japan). HLA alleles were automatically assigned using WAKFlow typing software (Wakunaga, Hiroshima, Japan). The probe sequences of the WAKFlow Typing kit are specifically designed to make allele determination easier in the Japanese population. By default, the analysis with WAKFlow typing software is based on the allele frequencies of the donors registered with Japan Marrow Donor Program (JMDP; https://www.bs.jrc.or.jp/bmdc/). This method can determine alleles with frequencies of ≥0.1% in the Japanese population.

## 6 Patient perspective

Acute hepatitis in children with unknown causes should be evaluated not only from the perspective of the virus, but should also host factors, including the HLA type.

## Data Availability

The datasets presented in this study can be found in online repositories. The names of the repository/repositories and accession number(s) can be found in the article/**Supplementary Material**.
