# Corrigendum: Case Report: HLA-DRB1 04:01 found in a child with adenovirus type 2 -linked hepatitis

**DOI:** 10.3389/fimmu.2025.1592744

**Published:** 2025-04-16

**Authors:** Yoshiki Katsumi, Yui Nishimura, Sachiko Goto, Seiichiro Ozawa, Tomoko Nishiura, Akira Kotera, Yoshiyuki Kawahara, Shiori Higashikawa, Rina Iwasaki, Yutaka Toriiminami, Norio Asai, Naohisa Fujita

**Affiliations:** ^1^ Department of Pediatrics, Kyoto Saiseikai Hospital, Kyoto, Japan; ^2^ Otokuni Health Center, Kyoto, Japan; ^3^ Kyoto Prefectural Institute of Public Health and Environment, Kyoto, Japan

**Keywords:** hepatitis, child, HLA-DR, adenovirus, common cold

In the published article, there was an error in the **Funding** statement. The grant number was incorrectly written as “Pfk0108636”. The correct **Funding** statement appears below.

“The author(s) declare financial support was received for the research, authorship, and/or publication of this article. The metagenomic analysis in this study was supported by AMED under Grant Number, JP22fk0108636.”

The authors apologize for this error and state that this does not change the scientific conclusions of the article in any way. The original article has been updated.

