# Supplementary material for: Case Report: HLA-DRB1 04:01 found in a child with adenovirus type 2 -linked hepatitis
Source: Front Immunol. 2025 Feb 26;16:1544622. doi: 10.3389/fimmu.2025.1544622 (PMC11896997; doi:10.3389/fimmu.2025.1544622)
Supplement: Supplementary file 1 [file DataSheet1.pdf]

Supplementary Figure 1(A)

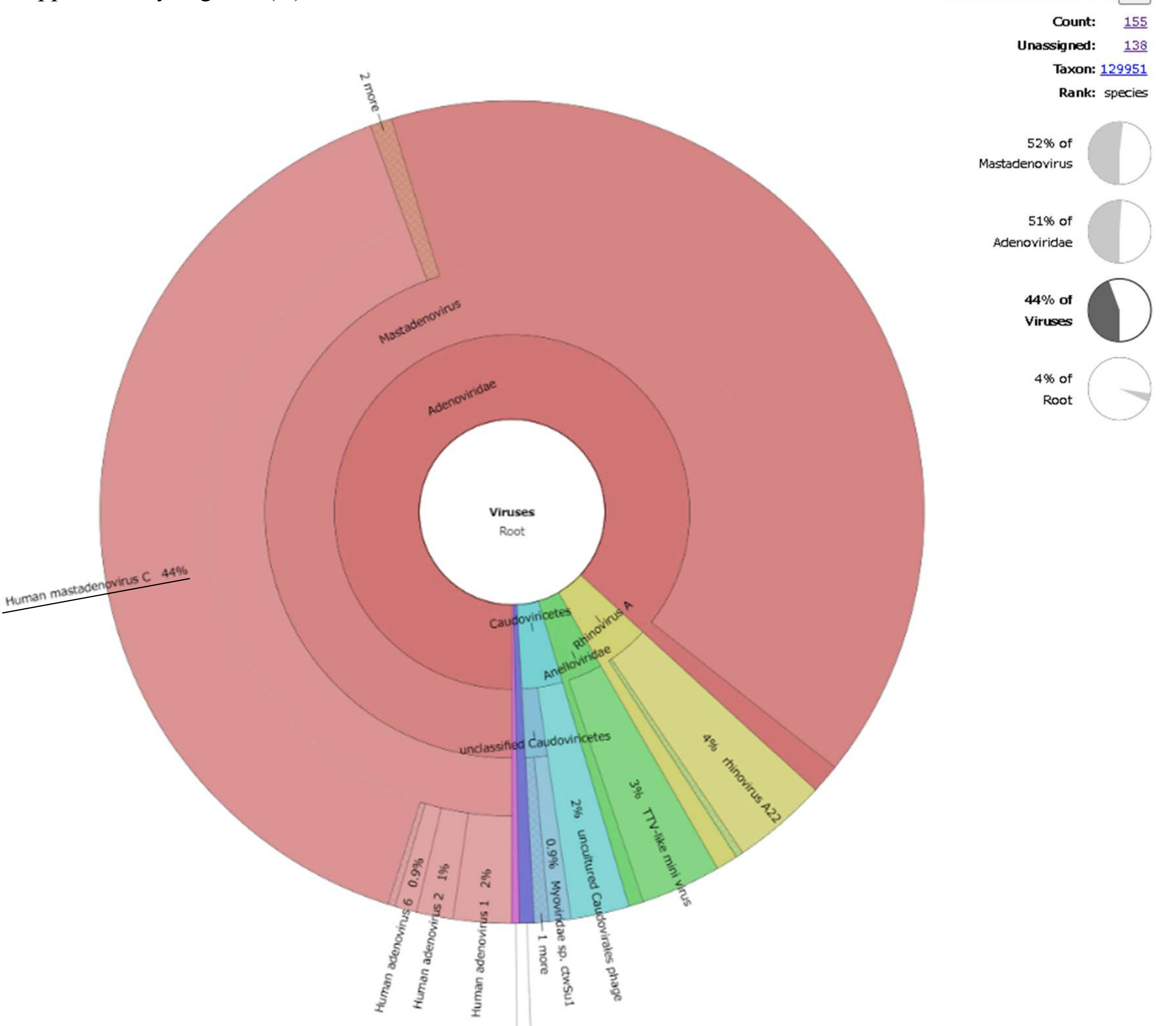

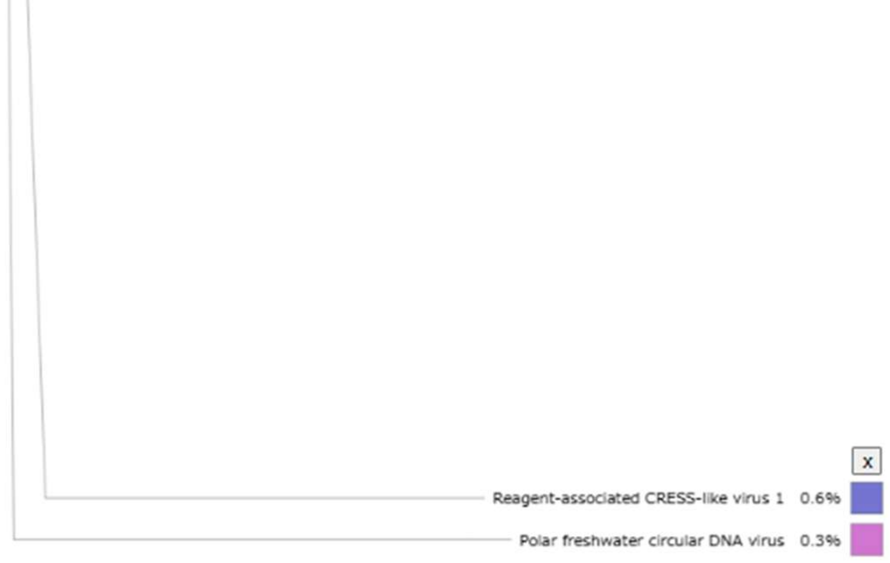

Metagenomic analysis in pharyngeal swab: Upper-right figure shows number of detected adenovirus reads and overall percentage of adenoviruses output by krona in the pharynx specimen. The root is the whole after removal of the human genome. The virus root is extracted in center of this figure. No pathogens other than human mastadenovirus C were detected that could cause hepatitis.

Supplementary Figure 1(B)

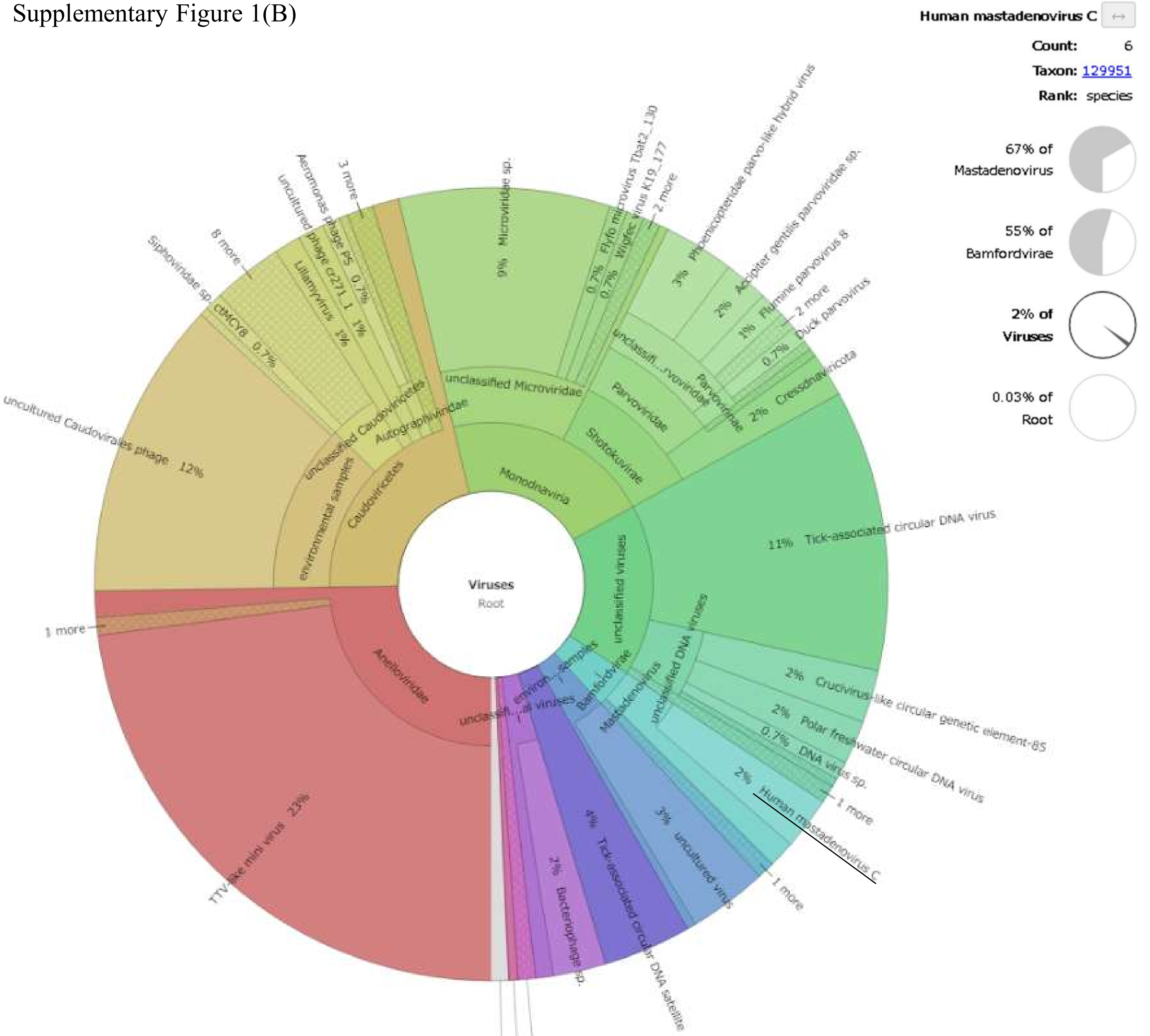

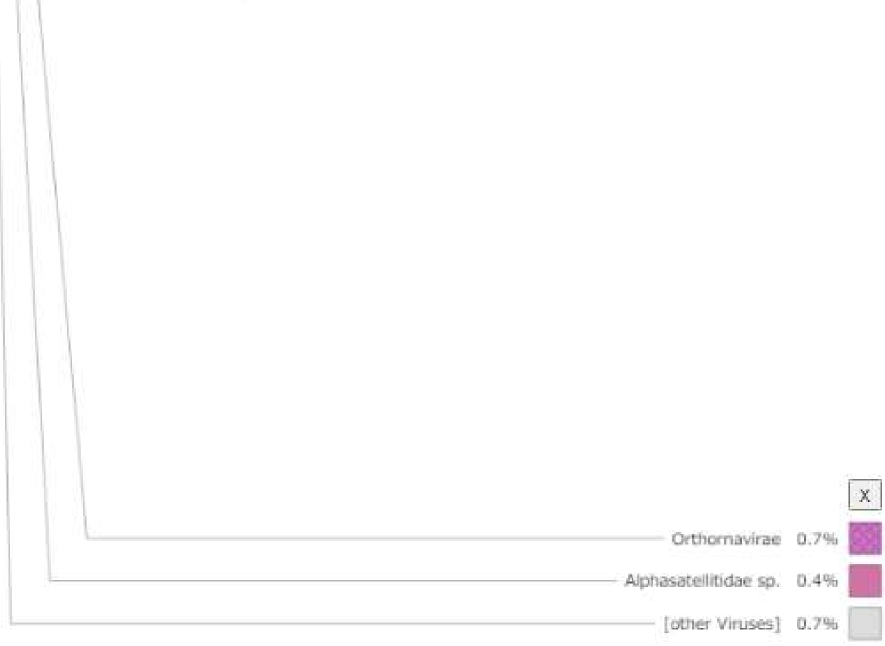

Metagenomic analysis in blood:
Upper-right figure shows number of detected adenovirus reads and overall percentage of adenoviruses output by krona in the blood specimen. The root is the whole after removal of the human genome. The virus root is extracted in center of this figure. No pathogens other than human mastadenovirus C were detected that could cause hepatitis.

Supplementary Figure 1(C)

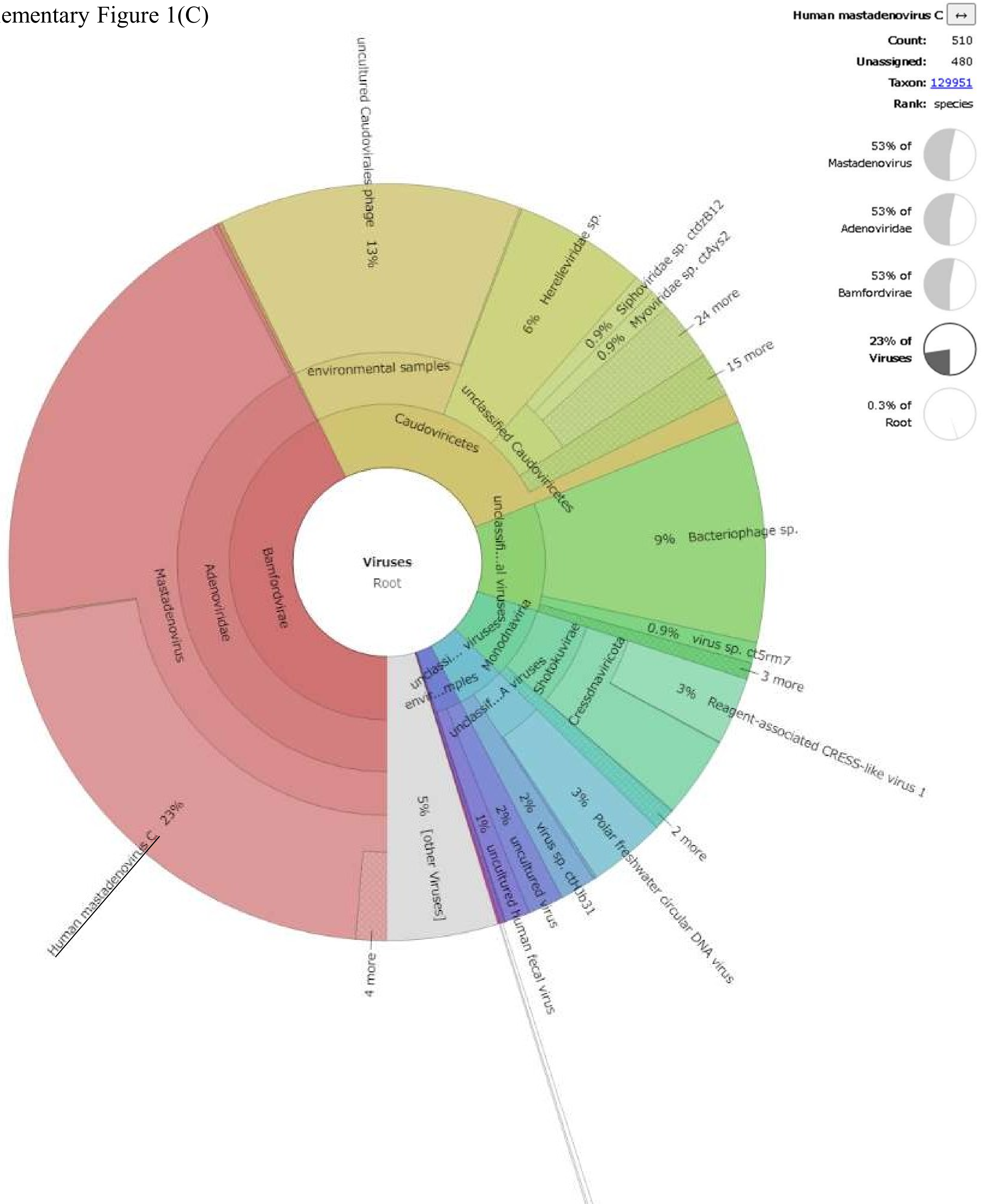

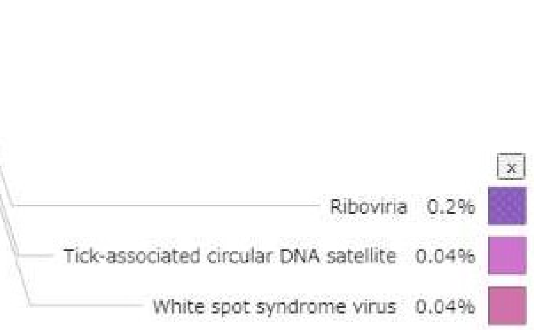

Metagenomic analysis in stool:
Upper-right figure shows number of detected adenovirus reads and overall percentage of adenoviruses output by krona in the stool specimen. The root is the whole after removal of the human genome. The virus root is extracted in center of this figure. No pathogens other than human mastadenovirus C were detected that could cause hepatitis.

Supplementary Figure 1(D)

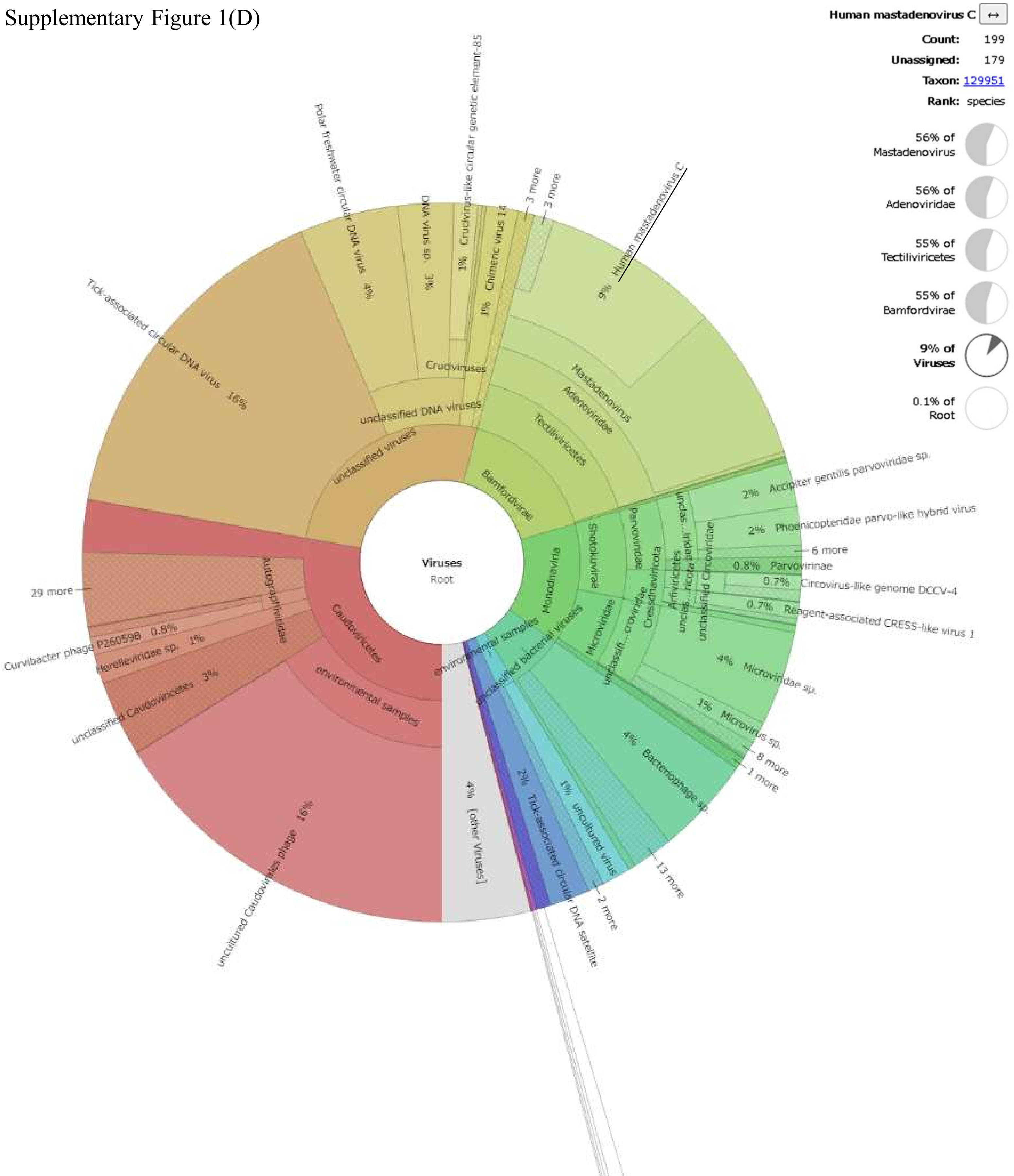

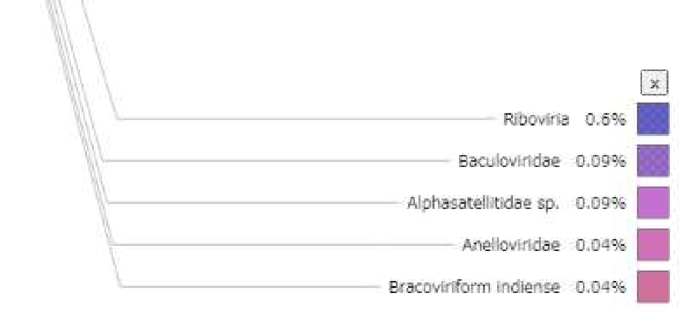

Metagenomic analysis in urine:
Upper-right figure shows number of detected adenovirus reads and overall percentage of adenoviruses output by krona in the urine specimen. The root is the whole after removal of the human genome. The virus root is extracted in center of this figure. No pathogens other than human mastadenovirus C were detected that could cause hepatitis.
