# Supplementary material for: Case Report: HLA-DRB1 04:01 found in a child with adenovirus type 2 -linked hepatitis
Source: Front Immunol. 2025 Feb 26;16:1544622. doi: 10.3389/fimmu.2025.1544622 (PMC11896997; doi:10.3389/fimmu.2025.1544622)
Supplement: Supplementary file 2 [file DataSheet2.pdf]

Supplementary Table 1: PCR testing to detect viruses that may cause acute hepatitis

	Pathogen	Results
Blood	Enterovirus	negative
	Parechovirus	negative
	Herpes simplex virus type 1	negative
	Herpes simplex virus type 2	negative
	Cytomegalovirus	negative
	Varicella zoster virus	negative
	Human betaherpesvirus type 6	negative
	Human betaherpesvirus type 7	negative
	Adenovirus type 2	positive
Stool	Enterovirus	negative
	Sapovirus	negative
	Norovirus	negative
	Rotavirus	negative
	Adenovirus type 2	positive
Pharyngeal swab	Enterovirus	negative
	Influenza virus	negative
	Severe acute respiratory syndrome coronavirus 2	negative
	Adenovirus type 2	positive
